# Chronic high-altitude exposure is associated with an internally-directed perceptual shift: differential links with attention networks and autonomic regulation

**DOI:** 10.3389/fnins.2026.1901035

**Published:** 2026-08-17

**Authors:** Xia-Qian Jian, Nian-Nian Wang, Xiao-Yan Huang, Rui Su, Hao Li, Jing Zhou, Hai-Lin Ma, Ming Liu, De-Long Zhang

**Affiliations:** 1 School of Psychology, Center for Studies of Psychological Application, and Guangdong Key Laboratory of Mental Health and Cognitive Science, South China Normal University, Guangzhou, China; 2 Xizang Key Laboratory of Plateau Hypoxic Environment and Life Health, Xizang University, Lhasa, China; 3 Anhui Provincial College Student Mental Health Education Research Center, School of Humanities and Management, Wannan Medical College, Wuhu, China; 4 School of Educational Sciences, Kashi University, Kashi, China

**Keywords:** attention networks, heart rate variability, high-altitude adaptation, internally-directed processing, perceptual resource allocation

## Abstract

**Background:**

High-altitude environments impose unique physiological and cognitive demands that may reorganize brain–body communication. Our study investigates whether chronic high-altitude exposure is associated with a shift in perceptual priority toward internally-directed processing, rather than merely modifying external processing speed or accuracy.

**Methods:**

We recruited 112 Han immigrants residing at high altitude to complete assessments of visual imagery vividness under eyes-open (externally-oriented) and eyes-closed (internally-oriented) conditions, along with attention network tests. We also collected a comprehensive panel of multimodal physiological indices, including markers of inflammation, erythrocytic parameters, liver function, cardiovascular metrics, and heart rate variability (as a proxy for autonomic regulation). Analyses employed latent profile analysis and regression mixture modeling.

**Results:**

We found that prolonged high-altitude exposure showed a trend toward selectively enhancing internally-oriented imagery vividness under eyes-closed conditions, while externally-oriented processing remained stable. Critically, enhanced internally-oriented imagery was differentially associated with improved orienting attention, whereas externally-oriented imagery was linked to alerting attention, suggesting a functional differentiation in attentional reorganization. Physiologically, interleukin-6 levels were associated with externally-oriented vividness, whereas erythrocytic heterogeneity, liver function, and high-frequency heart rate variability (an index of parasympathetic-associated cardiac regulation) selectively correlated with internally-oriented processing.

**Conclusion:**

These results suggest that chronic high-altitude exposure may be associated with a reweighting of perceptual resource allocation toward an internally-oriented processing bias, with parasympathetic-associated autonomic regulation as a potential brain–body integrative mechanism. This work extends prior findings by proposing a shift in perceptual priority rather than merely processing efficiency, advancing a multi-system framework for understanding human brain–body adaptation in extreme environments.

## 1 Introduction

High-altitude hypoxia represents a natural extreme environment that profoundly reshapes physiological homeostasis and cognitive functioning (Singh et al., 2004; Qin et al., 2020; Xue et al., 2022; Su et al., 2024). A growing body of research has revealed contrasting patterns of cognitive adaptation in high-altitude populations (Huang et al., 2023, 2025; Wang et al., 2023). For instance, Han immigrants with long-term high-altitude exposure often exhibit selective alterations in cognitive performance, such as reduced executive control or working memory (Griva et al., 2017; Ma et al., 2018, 2019; Li et al., 2021); paradoxically, they also reflect faster access to visual awareness, lower decision thresholds, and higher heart rate – a profile indicative of an efficiency-oriented, speed-prioritized strategy for external perceptual processing (Wang et al., 2023; Yu et al., 2023). In contrast, indigenous Tibetans display a “slow but steady” energy-saving attentional style characterized by reduced late neural activity, lower theta power, and greater reliance on motor-related networks to maintain high accuracy with minimal metabolic cost (Huang et al., 2025). Despite these advances, existing high-altitude cognitive research remains limited to investigating changes in exteroceptive processing efficiency, such as response speed, accuracy, or neural resource economy, while largely overlooking a more fundamental adaptive mechanism: whether chronic hypoxia reconfigures the priority and resource allocation between internal bodily signals and external sensory inputs (Marx et al., 2003, 2004; Taylor et al., 2010; Costumero et al., 2020; Park and Tallon-Baudry, 2014; Seth, 2013).

.Internally-directed processing refers to cognitive operations that are decoupled from immediate external sensory input and oriented toward internally generated representations, including visual mental imagery, autobiographical memory, and self-referential thought (Marx et al., 2003, 2004; Smallwood et al., 2012; Costumero et al., 2020). This mode of processing is supported by the default mode network (DMN) and is associated with distinct patterns of brain–body communication compared to externally-oriented processing, which is anchored in immediate sensory input (Raichle, 2015; Christoff et al., 2016). In the context of high-altitude adaptation, the interplay between these two modes may reflect a fundamental aspect of perceptual resource allocation that has been overlooked in prior research focused exclusively on processing efficiency. Importantly, closing one’s eyes provides a validated experimental manipulation that reduces external visual input and facilitates internally-directed cognition (Marx et al., 2003, 2004; Zhang et al., 2015; Costumero et al., 2020). While eyes-closed states do not equate to interoception in the narrow sense of visceral signal perception (e.g., heartbeat detection), they do create conditions in which mental imagery generation relies more heavily on internal resources rather than external sensory scaffolding.

The vividness of visual imagery differs significantly between the eyes-open and eyes-closed conditions, which offers an indirect avenue for investigating internal and external perceptual processing (Marx et al., 2003, 2004; Zhang et al., 2015; Costumero et al., 2020; Huang et al., 2023). Combining with the Attention Network Test (ANT), enables us to test the hypothesis that distinct attentional networks – specifically, alerting, orienting, and executive control – are differentially engaged to support interoceptive versus exteroceptive resource allocation (Fan et al., 2002, 2005; Cobos et al., 2019; Huang et al., 2023). We therefore hypothesized that long-term high-altitude exposure may be associated with a systematic reweighting of perceptual priorities, characterized by a differential association pattern: enhanced internally-directed processing would be specifically associated with improved orienting attention and modulated by parasympathetic-associated cardiac regulation, liver function, and erythrocytic parameters; whereas externally-oriented processing would remain stable and be linked to alerting attention and associated with inflammatory markers such as interleukin-6 (Bahrami et al., 2019; Balter et al., 2019).

The present study extends prior efficiency-focused frameworks through an internally-directed perceptual shift among Han immigrants. We employed a multimodal assessment battery – including eyes-open/closed imagery vividness, the Attention Network Test, and a comprehensive panel of physiological indices (e.g., autonomic, inflammatory, hematological, and hepatic markers) – to capture the interplay between perceptual reweighting and its underlying systemic drivers (Stein, 2002; Janszky et al., 2004; Lampert et al., 2008; Sun et al., 2016; Shaffer and Ginsberg, 2017; Bialuk et al., 2019; Gianfranchi et al., 2020; Ribeiro-Santos et al., 2020). Using latent profile analysis and regression mixture models, we tested the central hypothesis that the duration of chronic high-altitude exposure predicts a systematic shift toward an interoception-dominant perceptual mode (Magidson and Vermunt, 2002; Nylund et al., 2007; Vermunt, 2010). Collectively, this study offers a novel perspective on high-altitude adaptation by moving beyond traditional metrics of processing speed or energy conservation, and instead reveals a brain–body strategy centered on the dynamic reweighting of perceptual priorities.

## 2 Materials and methods

### 2.1 Participants

We recruited 112 healthy adult participants (58 males, 54 females; mean age = 35.0 ± 3.55 years) residing in Lhasa, Xizang Autonomous Region, China. All participants were Han Chinese who were born and raised in low-altitude regions (<1,500 m above sea level) and had relocated to the high-altitude environment (∼3,680 m) for durations ranging from 5 to 15 years (mean = 8.03 ± 2.78 years). Exclusion criteria included self-reported history of neurological or psychiatric disorders, regular medication use, or any medical conditions that could affect cardiovascular or cognitive functioning. All participants had normal or corrected-to-normal vision and were native Chinese speakers with comparable educational backgrounds. Written informed consent was obtained from all participants prior to study participation, and they received monetary compensation (¥500) for their time and effort.

### 2.2 Experimental design and procedure

**FIGURE 1:**
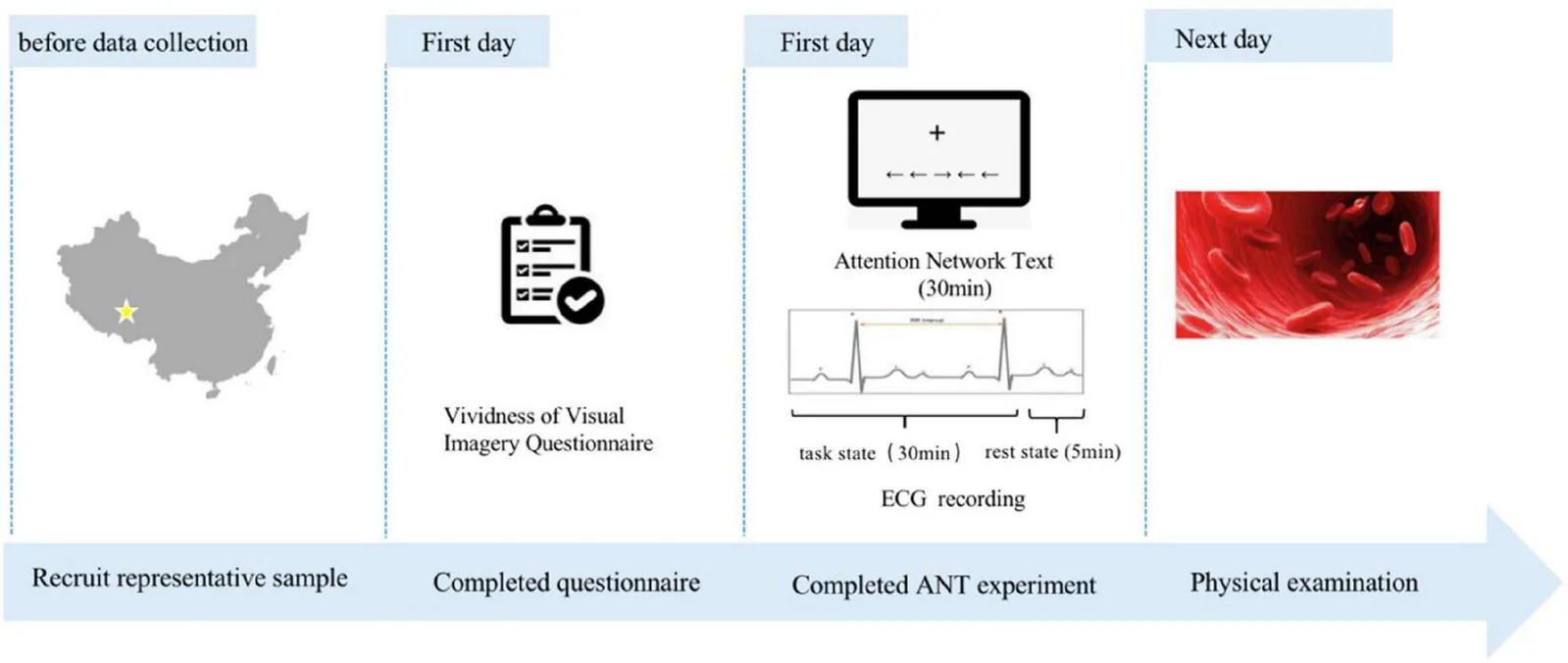
Schematic of experiment and data analysis.

The study employed a multi-session experimental design conducted over two consecutive days. On Day 1, participants first completed the Vividness of Visual Imagery Questionnaire (VVIQ) (Marks, 1973). To dissociate interoceptive and exteroceptive processing, the VVIQ was administered twice: once with eyes open and once with eyes closed, in a counterbalanced order across participants. Subsequently, participants completed ANT (Fan et al., 2002) while simultaneous electrocardiography was continuously recorded to derive heart rate variability metrics for analysis. On Day 2, participants underwent comprehensive physical examinations at the Fukang Physical Examination Center in Lhasa, including blood sample collection for biochemical analysis. The complete experimental timeline is illustrated in Figure 1.

### 2.3 Data acquisition

#### 2.3.1 Visual imagery vividness assessment

Visual imagery vividness was assessed using VVIQ (Marks, 1973), which comprises 16 items organized into four scenes (figure, sunrise, structure, and scenery) with four items per scene. Participants were instructed to generate mental images corresponding to each item description and rate the vividness on a 5-point scale ranging from 1 (perfectly clear and vivid, as if experiencing it directly) to 5 (no image at all, only thinking about the concept). Higher total scores indicate lower imagery vividness. The VVIQ was administered twice in fixed order: first under eyes-open conditions to assess externally-oriented processing, and subsequently under eyes-closed conditions to assess internally-directed processing. This administration sequence was maintained across all participants to ensure consistency in the assessment of state-dependent imagery processes.

#### 2.3.2 High-altitude exposure duration

Chronic high-altitude exposure duration was operationally defined as the cumulative time participants had resided continuously in the high-altitude environment (∼3,680 m), excluding temporary absences for vacation, work-related travel, or other short-term departures to low-altitude regions. This measure specifically captured sustained physiological adaptation to chronic hypoxic conditions.

#### 2.3.3 ANT

Attention network efficiency was assessed using ANT (Fan et al., 2002; Fan et al., 2005) implemented in MATLAB R2013a. The task employed a 3 (cue type: no cue, center cue, spatial cue) × 2 (target congruency: congruent, incongruent) factorial design. Each trial began with a central fixation cross (+) presented for a variable duration (400–1600 ms), followed by one of three cue conditions: no cue (fixation only), center cue (asterisk at fixation), or spatial cue (asterisk appearing 0.9° above or below fixation). After a 400-ms cue-target interval, the target stimulus appeared, consisting of five horizontally aligned arrows with the central arrow flanked by two arrows on each side. In congruent trials, all arrows pointed in the same direction; in incongruent trials, the flanking arrows pointed opposite to the central arrow. Participants responded to the direction of the central arrow by pressing “F” for leftward and “J” for rightward responses using their index fingers. Stimuli were presented in black against a gray background on a 19-inch monitor positioned 60 cm from the participant.

**FIGURE 2:**
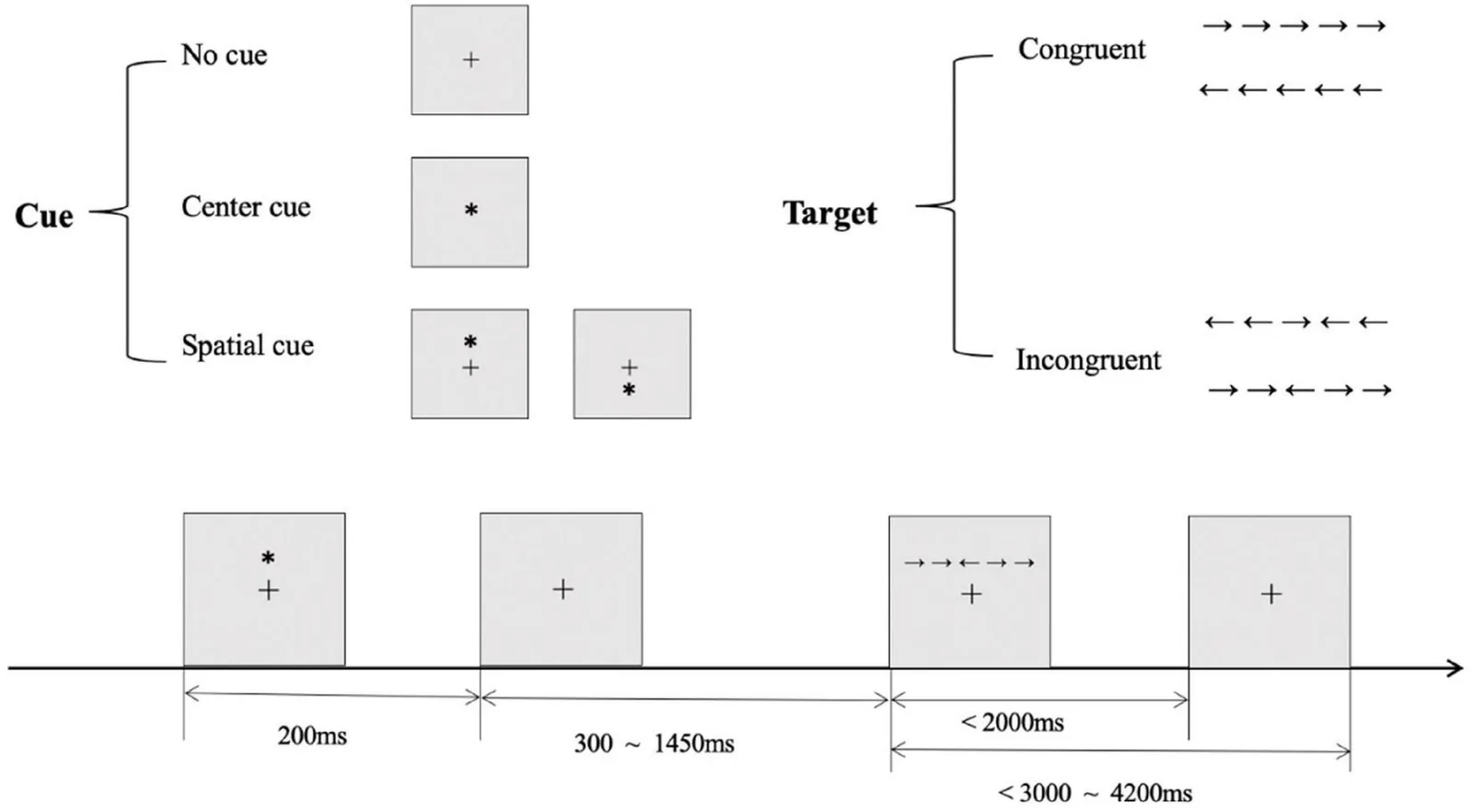
Schematic of the ANT procedure.

The experiment consisted of six blocks of 18 trials each (108 total trials), with each block containing equal proportions of the six experimental conditions. Prior to the main task, participants completed a full practice block to ensure task comprehension. Attention network efficiency scores were calculated from reaction times (RTs) using the following formulas: Alerting effect = RTno cue − RTcenter cue, Orienting effect = RTcenter cue − RTspatial cue, and Executive effect = RTincongruent − RTcongruent. The experimental paradigm is illustrated in Figure 2.

#### 2.3.4 Electrocardiographic recording and heart rate variability analysis

Electrocardiographic recordings were acquired using a BIOPAC MP150 system with AcqKnowledge^®^ software (version 4.4). Participants were tested individually in a quiet, temperature-controlled laboratory while seated comfortably in front of a computer monitor. Prior to electrode placement, skin sites were cleaned with alcohol and dried to minimize impedance. A modified lead II configuration was employed for optimal R-wave detection: the positive electrode was placed at the fifth intercostal space along the left midclavicular line (apex), the negative electrode at the right clavicular process (manubrium), and the ground electrode on the right abdomen. Electrocardiographic signals were recorded continuously at a sampling rate of 1,000 Hz throughout the experimental session.

Heart rate variability analysis was conducted using AcqKnowledge 4.2.1 software following established guidelines (Task Force of the European Society of Cardiology & North American Society of Pacing and Electrophysiology, 1996). Short-term heart rate variability was analyzed across three distinct 5-min epochs: (1) baseline rest period (first 5 min of recording), (2) task engagement period (final 5 min during ANT performance), and (3) post-task recovery period (5 min following task completion). Frequency domain analysis partitioned heart rate oscillations into standard bands: very-low-frequency (VLF: 0–0.04 Hz), low-frequency (LF: 0.04–0.15 Hz), and high-frequency (HF: 0.15–0.40 Hz) components (Rajendra Acharya et al., 2006; Shaffer et al., 2014). For the primary analysis examining associations between heart rate variability and perceptual processing, given our interest in the dynamic interplay between autonomic regulation and cognitive engagement, we focused our analysis on heart rate variability metrics derived from the ANT task period and the subsequent recovery period, baseline resting-period heart rate variability was adopted as the initial value of the steady state (Wang et al., 2014; Shaffer and Ginsberg, 2017; Nicolini et al., 2024).

#### 2.3.5 Blood sample collection and biochemical analysis

Comprehensive blood samples were collected on Day 2 at the Fukang Physical Examination Center in Lhasa following an overnight fast. Biochemical analyses included inflammatory markers (interleukin-6, high sensitivity C-reactive protein), homocysteine levels, liver function parameters (alanine aminotransferase, aspartate aminotransferase, total bilirubin), and complete blood count with erythrocytic indices (hemoglobin concentration, hematocrit, red blood cell count, mean corpuscular volume). All assays were performed using standardized clinical laboratory protocols with appropriate quality control measures.

### 2.4 Heart rate variability metrics and physiological interpretation

Heart rate variability serves as a sensitive index of neurocardiac function, reflecting dynamic interactions between the heart and brain through autonomic nervous system processes (Shaffer and Ginsberg, 2017; Nicolini et al., 2024; Task Force of the European Society of Cardiology & North American Society of Pacing and Electrophysiology, 1996). Optimal heart rate variability levels are associated with enhanced self-regulatory capacity and psychological resilience, whereas mental stress typically activates sympathetic nervous system activity, resulting in increased heart rate and decreased heart rate variability (Rajendra Acharya et al., 2006; Billman et al., 2015; Shaffer and Ginsberg, 2017). Based on established physiological interpretations, we focused on specific frequency bands relevant to our research questions: high frequency power reflects respiratory sinus arrhythmia and parasympathetic (vagal) modulation, making it particularly relevant for assessing cardiac regulation during cognitive task engagement; low frequency power is regulated by the baroreflex and represents the combined activity level of the sympathetic and parasympathetic nervous systems (Goldstein et al., 2011; Nicolini et al., 2024); and very low frequency power may reflect thermoregulatory, renin–angiotensin, and endothelial influences on cardiovascular function, with reduced very low frequency power associated with chronic inflammation (Akselrod et al., 1981; Stein, 2002; Janszky et al., 2004; Claydon and Krassioukov, 2008; Lampert et al., 2008; Shaffer et al., 2014; Usui and Nishida, 2017).

### 2.5 Data analysis

#### 2.5.1 Latent profile analysis and regression mixture modeling

Latent profile analysis was conducted to identify empirically derived subgroups based on visual imagery vividness patterns across the four VVIQ scenes (figure, sunrise, structure, scenery). Model selection proceeded incrementally, beginning with a 2-class solution and increasing the number of classes until model fit indices indicated no further improvement. Model fit was evaluated using multiple criteria: Akaike Information Criterion (AIC), Bayesian Information Criterion (BIC), and adjusted BIC (aBIC), where lower values indicate better fit; as well as entropy, where values closer to 1.0 indicate higher classification accuracy (Nylund et al., 2007). Additionally, the Lo-Mendell-Rubin adjusted likelihood ratio test (LMR-LRT) and bootstrap likelihood ratio test (BLRT) were employed to compare nested models, with *p* < 0.05 indicating significant improvement in model fit with the addition of another class.

Following identification of the optimal latent profile solution, regression mixture modeling was implemented using the R3STEP approach in Mplus 8.0 to examine predictors of class membership while accounting for classification uncertainty (Vermunt, 2010; Sun et al., 2016). This three-step procedure first estimates the measurement model, then assigns posterior probabilities, and finally regresses class membership on predictor variables (inflammatory markers, erythrocytic indices, liver function parameters, cardiovascular indicators, and high-altitude exposure duration) while correcting for classification uncertainty in the latent class assignment.

#### 2.5.2 Statistical analysis

**FIGURE 3:**
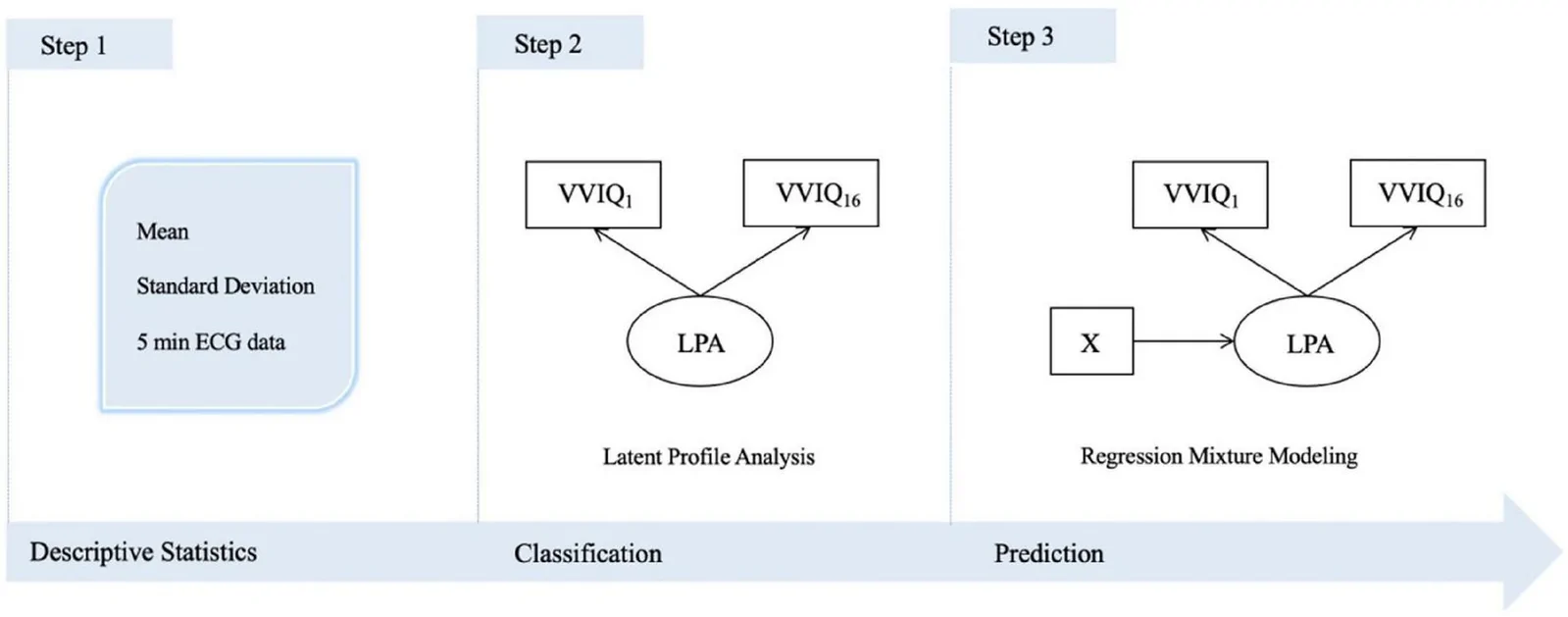
Schematic of data analysis. VVIQ_1_∼VVIQ_16_, variable; X, predictor variable; LPA, latent class variable.

All statistical analyses were conducted using SPSS 26.0 (IBM Corp.) and Mplus 8.0 (Muthén & Muthén). Descriptive statistics (means, standard deviations, frequencies) were computed for demographic, physiological, and behavioral variables. To compare perceptual vividness between the two eye-state conditions (a within-subjects factor), we employed a paired-samples permutation test (10,000 permutations). For comparisons of physiological and attentional variables across the empirically derived latent profile classes (a between-subjects factor), independent-samples permutation tests (10,000 permutations) were used. These non-parametric approaches were chosen to account for potential violations of normality assumptions in our multidimensional dataset (Nichols and Holmes, 2002; Zhang et al., 2014), providing robust inference without requiring strict distributional assumptions. This approach provides robust inference without requiring distributional assumptions while maintaining adequate statistical power. The complete analytical workflow is illustrated in Figure 3.

## 3 Results

### 3.1 Descriptive statistics

Descriptive statistics for demographic characteristics and biochemical indices (Table 1). The sample comprised 112 participants (58 males, 54 females; mean age = 35.0 ± 3.55 years) with high-altitude exposure durations ranging from 5 to 15 years (mean = 8.03 ± 2.78 years). Most biochemical parameters fell within normal clinical ranges, with the exception of interleukin-6, which had 30 missing values (26.8% missing rate). Missing data patterns were examined using Little’s MCAR test, which indicated that the missingness was consistent with a missing completely at random (MCAR) mechanism (χ^2^ = 12.34, *p* = 0.192). Accordingly, and for analytical simplicity, complete case analysis was performed in models including interleukin-6. However, it should be noted that this approach reduced the effective sample size for these specific analyses, which may impact statistical power. Consequently, complete case analysis was deemed appropriate for subsequent statistical modeling.

**TABLE 1:** Demographic characteristics and biochemical indices.

Variable	Mean (SD)	Variable	Mean (SD)
Age (years)	34.55 (3.5)	*Blood routine*	
gender	–	WBC	6.29 (1.6)
Immigrant time	8.03 (2.7)	NEUT#	3.39 (1.3)
		NEUT%	53.08 (8.5)
*Inflammation*		LYMPH#	2.31 (0.6)
hs-CRP	0.66 (0.8)	LYMPH%	37.19 (7.7)
IL-6	1.90 (7.4)	MONO#	0.44 (0.1)
		MONO%	7.20 (1.9)
*Stress response*		EO	0.13 (0.1)
HCY	17.97(15.0)	EO%	3.06 (9.5)
		BASO	0.02 (0.02)
*Liver functions*		BASO%	0.33 (0.3)
ALT	29.89 (26.9)	RBC	5.49 (0.6)
AST	24.39 (11.6)	HGB	167.60 (18.3)
GGT	38.38 (37.5)	HCT	47.76 (4.6)
ALP	68.55 (22.5)	MCV	87.08 (4.9)
TP	69.02 (3.7)	MCH	30.53 (2.0)
ALB	47.34 (2.8)	MCHC	350.58 (9.4)
GLB	21.68 (3.5)	RDW-SD	42.16 (2.8)
A/G	2.24 (0.4)	RDW-CV	13.44 (1.0)
TBIL	19.96 (8.0)	PLT	237.16 (60.3)
DBIL	5.39 (2.3)	PCT	0.26 (0.1)
UBIL	14.62 (5.9)	MPV	10.88 (1.0)
		PDW	13.42 (2.6)
		P-LCR	0.32 (0.1)

Immigrant time, the time of actual living in high-altitude area; hs-CRP, high sensitive C-reactive protein; IL-6, interleukin-6; HCY, homocysteine; ALT, alanine transaminase; AST, aspartate Transaminase; GGT, γ-glutamyl transpeptidase; ALP, alkaline phosphatase; TP, total protein; ALB, albumin; GLB, globulin; A/G, albumin/globulin; TBIL, total bilirubin; DBIL, direct bilirubin; UBIL, unconjugated bilirubin; TBA, total bile acid; WBC, white blood cell count; NEUT#, neutrophil count; NEUT%, neutrophil ratio; LYMPH#, lymphocyte count; LYMPH%, lymphocyte ratio; MONO#, monocyte count; MONO%, monocyte ratio; EO, eosinophil count; EO%, eosinophil ratio; BASO, basophil count; BASO%, basophil ratio; RBC, red blood cell count; HGB, hemoglobin; HCT, hematocrit; MCV, mean corpuscular volume; MCH, mean corpuscular hemoglobin; MCHC, mean corpuscular hemoglobin concentration; RDW-SD, standard deviation in red cell distribution width; RDW-CV, coefficient variation of red cell distribution width; PLT, platelet; PCT, thrombocytocrit; MPV, mean platelet volume; PDW, platelet distribution width; P-LCR, platelet larger cell ratio.

### 3.2 Latent profile analysis of visual imagery vividness

Latent profile analysis identified distinct subgroups characterized by differential visual imagery vividness patterns across eyes-open and eyes-closed conditions. Two separate latent class analyses were performed on VVIQ scores obtained under eyes-open and eyes-closed conditions, respectively. Each participant yielded 8 VVIQ indices, comprising 4 scenarios across the 2 viewing states. Model fit indices indicated that a 2-class solution was optimal for both the eyes-open and eyes-closed conditions (Table 2). The 2-class models demonstrated excellent classification accuracy, with entropy values of 0.963 eyes-open and 0.980 eyes-closed, indicating high certainty in class assignment. Additionally, both the Lo-Mendell-Rubin adjusted likelihood ratio test and bootstrap likelihood ratio test provided significant support for the 2-class over 1-class solutions (all *p* < 0.001).

**TABLE 2:** VVIQ Model fit statistics.

States	Classes	AIC	BIC	aBIC	Entropy	LMR, *p*	BLRT, *p*
Eyes-Open	1	5426.637	5513.629	5412.497	–	–	–
	2	4759.869	4893.076	4738.218	0.963	0.250	<0.00
	3	4424.158	4603.579	4394.996	0.938	0.360	<0.00
	4	4263.411	4489.047	4226.737	0.946	0.210	<0.00
Eyes-Closed	1	5614.469	5701.461	5600.330	–	–	–
	2	4913.637	5046.843	4891.986	0.980	0.286	<0.00
	3	4625.460	4804.881	4596.298	0.952	0.213	<0.00
	4	4347.514	4573.150	4310.840	0.959	0.066	<0.00

AIC, Akaike information criteria; BIC, Bayes information criteria; aBIC, adjusted Bayes information criteria; LMR, Lo-Mendell-Rubin; BLRT, Bootstrap Likelihood Ratio Test. *p* < 0.05 indicates improved fit compared to a solution with one class less.

**FIGURE 4:**
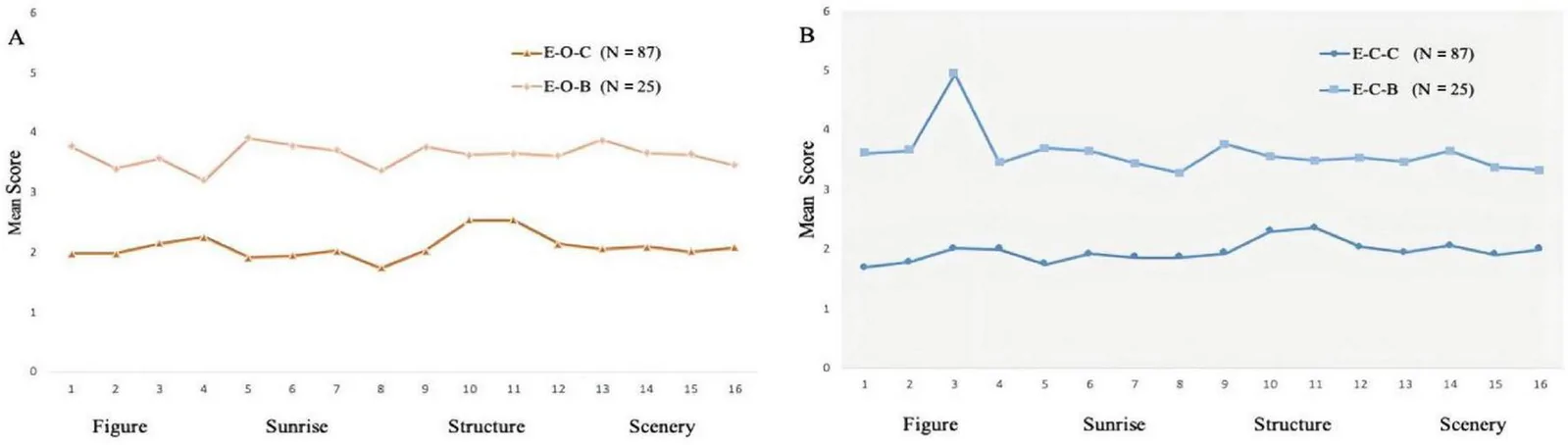
Latent profile analysis identified 2 profiles during eyes-open and eyes-closed states. (**A**) Pattern of VVIQ in eyes-open state; **(B)** Pattern of VVIQ in eyes-closed state; E-O-C, eyes-open-clear; E-O-B, eyes-open-blur; E-C-C, eyes-closed-clear; E-C-B, eyes-closed-blur.

Based on VVIQ scoring conventions (higher scores indicate lower imagery vividness), the two classes were labeled as follows: For eyes-open conditions, Class 1 (*n* = 87) represented “eyes-open clear” (E-O-C) imagery, while Class 2 (*n* = 25) represented “eyes-open blur” (E-O-B) imagery. Similarly, for eyes-closed conditions, Class 1 (*n* = 87) represented “eyes-closed clear” (E-C-C) imagery, and Class 2 (*n* = 25) represented “eyes-closed blur” (E-C-B) imagery. The distinct imagery vividness profiles for each class are illustrated in Figures 4A, B.

When sex was included as a covariate in the regression mixture model, the pattern of results remained largely unchanged. Sex itself did not significantly predict class membership (eyes-open: *p* = 0.471; eyes-closed: *p* = 0.471).

### 3.3 Effects of chronic high-altitude exposure duration

Regression mixture modeling examined whether chronic high-altitude exposure duration predicted visual imagery vividness class membership. In eyes-open conditions, exposure duration showed no significant association with imagery vividness classification (regression coefficient = −0.129, SE = 0.082, *p* = 0.131, OR = 0.879). Similarly, participant age did not significantly predict eyes-open imagery vividness (regression coefficient = −0.023, SE = 0.056, *p* = 0.681, OR = 0.977).

In contrast, under eyes-closed conditions, there was a trend approaching significance for a positive association between high-altitude exposure duration and eyes-closed clear classification. (regression coefficient = 0.179, SE = 0.098, *p* = 0.069, OR = 1.196). This suggests that longer residence in high-altitude environments was associated with a greater probability of belonging to the eyes-closed-clear group (clearer interoceptive imagery). Age remained non-significant in eyes-closed conditions (regression coefficient = 0.016, SE = 0.078, *p* = 0.830, OR = 1.016). These findings suggest a state-dependent effect of chronic high-altitude exposure, with a specifical trend toward enhancing interoceptive (eyes-closed) rather than exteroceptive (eyes-open) imagery vividness (Table 3).

**TABLE 3:** The predictor variable of potential category.

Predictor variable	R			R		
	E-O-C	E-O-B	*P*-value	E-C-C	E-C-B	*P*-value
Age	−0.023	0.023	0.681	0.016	−0.016	0.830
Immigrant time	−0.129	0.129	0.131	**0.179**	−**0.179**	0.069*

Immigrant time: the time of actual living in high-altitude area; E-O-C, eyes-open-clear; E-O-B, eyes-open-blur; E-C-C, eyes-closed-clear; E-C-B, eyes-closed-blur; R, Regression coefficient.

*p < 0.05,

**p < 0.01,

***p < 0.001. Bold values indicate significant associations.

### 3.4 Attention network differences between eye states

To test our hypothesis of a differential association pattern between attention networks and perceptual modes, we directly compared attention network efficiency scores between the imagery vividness groups identified in the latent profile analysis. Non-parametric permutation tests revealed a significant association between alerting efficiency and exteroceptive processing: participants in the eyes-open-clear group exhibited significantly higher alerting efficiency than those in the eyes-open-blur group (mean difference = 18.7 ms, *p* = 0.042, Cohen’s d = 0.42). For interoceptive processing, a parallel comparison showed that participants in the eyes-closed-clear group link to a trend toward higher orienting efficiency compared to the eyes-closed-blur group, though this effect did not reach conventional significance (mean difference = 22.3 ms, *p* = 0.069, Cohen‘s d = 0.38). No other significant differences were observed for the orienting (*p* = 0.329) or executive (*p* = 0.237) networks in eyes-open conditions, or for the alerting (*p* = 0.246) and executive (*p* = 0.484) networks in eyes-closed conditions (Table 4). Together, these findings provide preliminary evidence consistent with a differential association pattern, wherein alerting attention supports exteroceptive clarity, while orienting attention is specifically linked to interoceptive vividness.

**TABLE 4:** Attentional network scores based on the RT (*M* ± *SD*).

Attention networks	RT			RT		
	E-O-C	E-O-B	*P*-value	E-C-C	E-C-B	*P*-value
Alerting	5.66 ± 11.12	1.51 ± 10.27	0.042* *	4.44 ± 10.81	5.76 ± 11.92	0.246
Orienting	60.10 ± 20.30	58.02 ± 21.05	0.329	60.75 ± 21.64	55.78 ± 15.03	0.069*
Executive	95.03 ± 26.02	99.40 ± 34.69	0.237	96.07 ± 27.75	95.78 ± 29.74	0.484

RT, Reaction times (*M* ± *SD*); E-O-C, eyes-open-clear; E-O-B, eyes-open-blur; E-C-C, eyes-closed-clear; E-C-B, eyes-closed-blur.

*p < 0.05,

**p < 0.01,

***p < 0.001. Bold values indicate significant associations.

A mixed-design ANOVA with group (clear vs. blur) as the between-subjects factor and attention network (alerting, orienting, executive) as the within-subjects factor was conducted separately for eyes-open and eyes-closed classifications. For the eyes-open classification: main effect of group: F(1, 110) = 0.057, *p* = 0.811; main effect of attention network: F(2, 220) = 366.327, *p* = 0.001; Group × Attention network interaction: F(2, 220) = 0.817, *p* = 0.443. For the eyes-closed classification: main effect of group: F(1, 110) = 0.257, *p* = 0.613; main effect of attention network: F(2, 220) = 342.937, *p* = 0.001; Group × Attention network interaction: F(2, 220) = 0.440, *p* = 0.644.

### 3.5 Frequency domain heart rate variability analysis

Frequency domain analysis of heart rate variability revealed state-dependent associations with visual imagery vividness. In eyes-open conditions, no significant differences emerged between eyes-open-clear and eyes-open-blur groups across any heart rate variability frequency bands (all *p* > 0.05).

In eyes-closed conditions, however, specific heart rate variability components significantly predicted imagery vividness classification (Table 5). Given our interest in the dynamic interplay between autonomic regulation and cognitive engagement, we focused our analysis on heart rate variability metrics derived from the ANT task period and the subsequent recovery period, rather than the baseline rest period. During the 5 min of task performance, high-frequency power was marginally associated with eyes-closed-clear classification (regression coefficient = 0.148, SE = 0.061, *p* = 0.014, *q* = 0.07, OR = 1.160), indicating that greater parasympathetic (vagal) activity during cognitive engagement was associated with clearer interoceptive imagery.

**TABLE 5:** The predictor variable to potential category on frequency domain analysis.

Predictor variable	R			R			
	E-O-C	E-O-B	*P*-value	E-C-C	E-C-B	*P*-value	q (FDR-corrected)
Baseline rest period							
HR	−0.007	0.007	0.610	–0.014	0.014	0.311	0.778
HRV	0.046	–0.046	0.812	0.130	−0.130	0.438	0.438
VLF	−0.015	0.015	0.831	−0.065	0.065	0.346	0.577
LF	0.023	−0.023	0.764	−0.064	0.064	0.349	0.438
HF	0.006	−0.006	0.688	**0.137**	−**0.137**	0.057*	0.285
Task engagement period							
HR	−0.012	0.012	0.382	−0.020	0.020	0.191	0.318
HRV	0.035	−0.035	0.749	0.050	−0.050	0.650	0.650
VLF	−0.115	0.115	0.108	−**0.180**	**0.180**	0.040**	0.100
LF	−0.046	0.046	0.518	−0.073	0.073	0.258	0.3225
HF	0.059	−0.059	0.399	**0.148**	−**0.148**	0.014**	0.0700*
Post-task recovery period							
HR	−0.012	0.012	0.389	−0.020	0.020	0.208	0.347
HRV	−0.012	0.012	0.869	−0.092	0.092	0.150	0.375
VLF	−0.025	0.025	0.731	−**0.238**	**0.238**	0.002**	0.01**
LF	−0.060	0.06	0.362	0.022	−0.022	0.735	0.919
HF	−0.112	0.112	0.111	−0.010	0.010	0.892	0.892

HR, heart rate; HRV, heart rate variability; VLF, very low frequency; LF, low frequency; HF, high frequency; R, Regression coefficient; E-O-C, eyes-open-clear; E-O-B, eyes-open-blur; E-C-C, eyes-closed-clear; E-C-B, eyes-closed-blur.

*p < 0.05,

**p < 0.01,

***p < 0.001. Bold values indicate significant associations.

Conversely, very-low-frequency power showed inverse associations with eyes-closed imagery vividness. This pattern was more pronounced during the 5-min post-task rest period, where higher very-low-frequency power significantly predicted eyes-closed-blur classification (regression coefficient = −0.238, SE = 0.076, *p* = 0.002, q = 0.01, OR = 0.788). No significant differences emerged for heart rate, total heart rate variability, or low-frequency power between eyes-closed groups.

### 3.6 Physiological predictors of interoceptive imagery vividness

#### 3.6.1 Inflammatory markers

Interleukin-6 showed a marginally significant association with eyes-open imagery vividness, with eyes-open-clear participants exhibiting higher Interleukin-6 levels than eyes-open-blur participants (regression coefficient = 0.054, SE = 0.028, *p* = 0.053, OR = 1.056). To assess the robustness of the IL-6 finding, we conducted multiple imputation using 20 imputed datasets. The pooled estimates were consistent with the complete-case analysis: regression coefficient = 3.011, SE = 0.348, *p* = 0.003.

High-sensitivity C-reactive protein showed no significant association with eyes-open imagery vividness (*p* = 0.996). In eyes-closed conditions, neither Interleukin-6 (*p* = 0.789) nor High-sensitivity C-reactive protein (*p* = 0.889) significantly predicted imagery vividness classification (Table 6).

**TABLE 6:** The predictor variable of potential category.

Predictor variable	R			q (FDR-corrected)	R			q (FDR-corrected)
	E-O-C	E-O-B	*P*-value		E-C-C	E-C-B	*P*-value	
Inflammation								
hs-CRP	−0.002	0.002	0.996	0.996	0.042	−0.042	0.889	0.889
IL-6	**3.011**	−**3.011**	**0.003** **	**0.006** **	0.039	−0.039	0.789	1.578
Stress response								
HCY	−0.013	0.013	0.380	0.380	0.005	−0.005	0.656	0.656
Liver functions								
ALT	−0.004	0.004	0.677	0.772	−0.002	0.002	0.822	0.916
AST	0.011	−0.011	0.564	0.752	−0.002	0.002	0.916	0.916
GGT	0.006	−0.006	0.332	0.531	−0.001	0.001	0.847	0.916
ALP	0.002	−0.002	0.772	0.772	−0.006	0.006	0.542	0.867
TP	−0.108	0.108	0.062	0.496	−**0.258**	**0.258**	0.000***	0.000***
ALB	−0.073	0.073	0.275	0.550	−**0.188**	**0.188**	0.017**	0.0667*
GLB	−0.076	0.076	0.198	0.531	−**0.148**	**0.148**	0.025**	0.0667*
A/G	−0.067	0.067	0.246	0.656	0.745	−0.745	0.178	0.356
Blood routine								
WBC	−0.077	0.077	0.522	0.720	−0.115	0.115	0.365	0.663
NEUT#	−0.192	0.192	0.249	0.683	−0.188	0.188	0.251	0.568
NEUT%	−0.063	0.063	0.039	0.336	−0.045	0.045	0.078	0.384
LYMPH#	0.332	−0.332	0.393	0.720	0.101	0.101	0.788	0.860
LYMPH%	0.064	−0.064	0.058	0.348	0.054	−0.054	0.068	0.384
MONO#	**3.947**	−**3.947**	0.042*	0.336	−0.740	0.740	0.653	0.817
MONO%	**3.947**	−**3.947**	0.002**	0.048**	0.099	−0.099	0.387	0.663
EO	−1.429	1.429	0.453	0.720	−0.947	0.947	0.577	0.817
EO%	0.016	−0.016	0.345	0.720	0.021	−0.021	0.284	0.568
BASO	−14.502	14.502	0.256	0.683	−15.988	15.988	0.220	0.568
BASO%	− 0.839	0.839	0.312	0.720	−0.964	0.964	0.261	0.568
RBC	−0.186	0.186	0.570	0.720	−0.144	0.144	0.675	0.817
HGB	−0.006	0.006	0.635	0.726	−0.008	0.008	0.529	0.817
HCT	−0.034	0.034	0.475	0.720	−0.008	0.008	0.830	0.866
MCV	−0.005	0.005	0.941	0.941	0.016	−0.016	0.704	0.817
MCH	0.014	−0.014	0.851	0.928	−0.051	0.051	0.715	0.817
MCHC	0.013	−0.013	0.503	0.720	−0.045	0.045	0.096	0.384
RDW-SD	−0.113	0.113	0.113	0.452	**0.248**	−**0.248**	0.004**	0.096*
RDW-CV	−0.347	0.347	0.097	0.452	**0.649**	−**0.649**	0.013**	0.156
PLT	0.003	−0.003	0.544	0.720	−0.006	0.006	0.128	0.439
PCT	3.140	−3.140	0.500	0.720	0.158	−0.158	0.085	0.384
MPV	0.010	−0.010	0.933	0.941	0.001	−0.001	0.983	0.983
PDW	−0.040	0.040	0.607	0.726	−0.045	0.045	0.594	0.817
P-LCR	0.244	−0.244	0.180	0.617	0.239	−0.239	0.199	0.568

hs-CRP, high sensitive C-reactive protein; IL-6, interleukin-6; HCY, homocysteine; ALT, alanine transaminase; AST, aspartate Transaminase; GGT, γ-glutamyl transpeptidase; ALP, alkaline phosphatase; TP, total protein; ALB, albumin; GLB, globulin; A/G, albumin/globulin; WBC, white blood cell count; NEUT#, neutrophil count; NEUT%, neutrophil ratio; LYMPH#, lymphocyte count; LYMPH%, lymphocyte ratio; MONO#, monocyte count; MONO%, monocyte ratio; EO, eosinophil count; EO%, eosinophil ratio; BASO, basophil count; BASO%, basophil ratio; RBC, red blood cell count; HGB, hemoglobin; HCT, hematocrit; MCV, mean corpuscular volume; MCH, mean corpuscular hemoglobin; MCHC, mean corpuscular hemoglobin concentration; RDW-SD, standard deviation in red cell distribution width; RDW-CV, coefficient variation of red cell distribution width; PLT, platelet; PCT, thrombocytocrit; MPV, mean platelet volume; PDW, platelet distribution width; P-LCR, platelet larger cell ratio; R, Regression coefficient; E-O-C, eyes-open-clear; E-O-B, eyes-open-blur; E-C-C, eyes-closed-clear; E-C-B, eyes-closed-blur;

*p < 0.1;

**p < 0.05;

***p < 0.001. Bold values indicate significant associations.

#### 3.6.2 Homocysteine and visual imagery vividness

Homocysteine levels showed no significant association with visual imagery vividness in either eye state. In eyes-open conditions, homocysteine concentrations did not differ significantly between eyes-open-clear and eye-open-blur groups (mean difference = 0.8 μmol/L, *p* = 0.671, Cohen’s d = 0.12), and homocysteine failed to predict imagery vividness classification in regression analysis (*p* = 0.723). Similarly, in eyes-closed conditions, no significant group differences emerged for homocysteine (mean difference = 1.2 μmol/L, *p* = 0.127, Cohen’s d = 0.28), and regression analysis showed no predictive relationship (*p* = 0.156).

#### 3.6.3 Liver function parameters

Liver function parameters showed no significant associations with eyes-open imagery vividness (all *p* > 0.05). However, in eyes-closed conditions, multiple liver function markers significantly predicted imagery vividness classification. Higher total protein levels were associated with eyes-closed-blur classification (regression coefficient = −0.258, SE = 0.067, *p* < 0.001, *q* < 0.001, OR = 0.773). Similarly, higher albumin levels marginally predicted eyes-closed-blur classification after FDR correction (regression coefficient = –0.188, SE = 0.079, *p* = 0.017, *q* = 0.0667, OR = 0.829), as did higher globulin levels (regression coefficient = –0.148, SE = 0.066, *p* = 0.025, *q* = 0.0667, OR = 0.862). These findings suggest that higher circulating levels of liver-synthesized proteins (total protein, albumin, and globulin) are associated with reduced interoceptive imagery vividness.

#### 3.6.4 Erythrocytic indices

Most erythrocytic parameters showed no significant associations with eyes-open imagery vividness. However, monocyte ratio was significantly higher in eyes-open-clear participants compared to eyes-open-blur participants (regression coefficient = 3.947, SE = 1.923, *p* = 0.002, *q* = 0.048, OR = 51.98).

In eyes-closed conditions, red blood cell distribution width measures showed significant associations with imagery vividness. Both standard deviation in red cell distribution width (regression coefficient = 0.248, SE = 0.087, *p* = 0.004, OR = 1.281) and coefficient variation of red cell distribution width (regression coefficient = 0.649, SE = 0.256, *p* = 0.013, OR = 1.914) were significantly higher in eyes-closed-clear participants compared to eyes-closed-blur participants. This pattern suggests that greater erythrocytic heterogeneity is associated with enhanced interoceptive imagery vividness, potentially reflecting adaptive hematological responses to chronic hypoxic stress (Table 6).

## 4 Discussion

This study provides evidence suggesting that chronic high-altitude exposure may be associated with a shift in perceptual priority, with resources differentially allocated toward internally-directed processing. This pattern appears to be supported by differential associations with attention networks and modulated by a coordinated neuroimmune-autonomic axis. Below, we discuss these findings in the context of high-altitude adaptation, acknowledging that, given the cross-sectional design of the present study, all associations reported herein should be interpreted as correlational rather than causal. Longitudinal studies tracking individuals before and after relocation to high altitude are needed to establish experience-dependent adaptive plasticity.

### 4.1 High-altitude exposure toward interoceptive dominance

Our findings extend prior work on altitude-induced cognitive changes (Zhang et al., 2018; Ma et al., 2020; Zhou et al., 2024) by demonstrating a state-dependent reorganization of perceptual resources. While previous studies noted alterations in alerting or executive functions (Ma et al., 2020; Xue et al., 2022), our data suggest that the critical adaptation lies in the differential engagement of these functions based on perceptual context. Specifically, we observed a trend in which longer residence at high-altitude was associated with a greater likelihood of exhibiting clear vivid internally-directed imagery, while externally-oriented processing remained stable. This pattern supports the hypothesis that the high-altitude environment fosters an internally-directed bias in perceptual processing.

### 4.2 Conceptual considerations: internally-directed vs. interoceptive processing

A critical conceptual issue warrants explicit discussion. Throughout this manuscript, we use the terms “internally-directed” and “externally-oriented” to describe the direction of perceptual resource allocation under eyes-closed and eyes-open conditions, respectively. We deliberately avoid equating eyes-closed imagery with interoception in the strict sense of visceral signal perception (e.g., heartbeat detection or gastric awareness) (Critchley and Harrison, 2013). Closing one’s eyes reduces external visual input and may facilitate internally-directed cognition – including visual mental imagery, autobiographical memory retrieval, and default mode network activity – but this is not equivalent to the perception of interoceptive signals per se (Smallwood et al., 2012; Christoff et al., 2016).

Previous neuroimaging studies have established that eyes-open and eyes-closed states produce systematic differences in brain activity patterns, including modulation of the default mode network, visual cortex, and salience network (Marx et al., 2003; Marx et al., 2004; Zhang et al., 2015; Costumero et al., 2020). These differences support the use of eye state as a manipulation of perceptual orientation. However, we acknowledge that this operationalization captures a broader construct of internally-directed cognition rather than narrowly defined interoception. Future studies employing direct interoceptive measures – such as heartbeat counting tasks (Schandry, 1981), heartbeat evoked potentials (Park et al., 2014), or gastric myoelectric recordings – would provide more precise tests of whether high-altitude exposure specifically enhances interoceptive processing versus internally-directed cognition more broadly.

### 4.3 Differential associations between attention networks and perceptual modes

A key finding of our study is the differential association pattern between attention networks and perceptual modes. We observed that clearer externally-oriented imagery was significantly associated with enhanced alerting efficiency, while a trend linked clearer internally-oriented imagery to superior orienting efficiency. Within Posner‘s framework (Posner, 1994; Petersen and Posner, 2012), this pattern suggests that under chronic hypoxic stress, the cognitive system may differentially engage attention networks based on perceptual context: endogenous orienting may be recruited to manage internal representations, while exogenous alerting may be preserved for external vigilance.

We note, however, that our evidence for this differential pattern is based on separate between-group comparisons rather than a significant interaction effect in a formal omnibus test. The current findings should therefore be interpreted as suggestive of a functional differentiation rather than a confirmed double dissociation in the strict cognitive neuroscience sense (Davies, 2010). Future studies with larger samples and pre-registered interaction tests are needed to rigorously test this hypothesis.

### 4.4 The association between physiological mechanisms and visual imagery vividness

Our multimodal physiological analysis revealed a coordinated autonomic and hematological signature that differentially correlated with internally-directed and externally-oriented processing. Central to this pattern was heart rate variability, a widely used index of cardiac autonomic regulation (Shaffer and Ginsberg, 2017). We found that higher high-frequency heart rate variability – commonly interpreted as reflecting parasympathetic activity, though we note it is an indirect marker rather than a direct measure of vagal nerve function (Farmer et al., 2016) – during cognitive engagement was positively associated with clearer internally-oriented imagery. This finding suggests that parasympathetic-associated cardiac regulation may support the internal focus required for vivid mental imagery generation (Stein, 2002; Janszky et al., 2004; Lampert et al., 2008; Usui and Nishida, 2017; Jiang et al., 2020).

Hematological findings further reflect this adaptive landscape. Greater erythrocytic heterogeneity, as indexed by higher red cell distribution width standard deviation and coefficient of variation, was significantly associated with enhanced internally-directed imagery vividness. This pattern likely reflects an adaptive hematopoietic response to chronic hypoxia, where increased production and release of new erythrocytes enhance oxygen delivery capacity, thereby supporting the metabolic demands of internal simulation (Xue et al., 2022).

In contrast, the physiological profile associated with externally-oriented processing followed a different trajectory. Higher circulating levels of the pro-inflammatory cytokine interleukin-6 were associated with clearer imagery in the eyes-open state. While systemic inflammation is often implicated in global cognitive deficits (Bradburn et al., 2018), our finding points to a context-dependent role for specific inflammatory signals in perceptual prioritization under environmental stress, a phenomenon observed in other high-altitude studies (Wang et al., 2024; Xue et al., 2022). Importantly, this IL-6 finding should be regarded as preliminary. Although the association survived false discovery rate (FDR) correction and was robust to multiple imputation, the IL-6 data had a 26.8% missing rate, which warrants cautious interpretation and replication in larger samples. Interestingly, higher levels of liver-synthesized proteins (total protein, albumin, and globulin) were associated with reduced clarity in the eyes-closed state, a relationship that may warrant further investigation into the complex interplay between hepatic function, fluid balance, and cognitive resource allocation in hypoxic environments.

In sum, these results point to a potential multisystem framework, in which vagal activity and adaptive hematological changes are associated with internally-directed processing, while specific inflammatory signals are linked to the maintenance of vigilance toward the external environment, together supporting a dynamic and resilient mode of adaptation in extreme environments.

### 4.5 Implications, limitations, and future directions

Several important limitations should be acknowledged:

First, and most critically, our operationalization of internally-directed processing via eyes-closed VVIQ is an approximation rather than a direct measure. As discussed in Section “4.2 Conceptual considerations: internally-directed vs. interoceptive processing”, closing one’s eyes reduces external sensory input and may facilitate internally-directed cognition, but this does not equate to interoception in the strict sense. Future studies should employ direct interoceptive measures (heartbeat counting, heartbeat evoked potentials) to disentangle internally-directed imagery from visceral perception.

Second, the VVIQ was administered in a fixed order (eyes-open first, then eyes-closed) across all participants. While this follows standard VVIQ administration protocols, it may introduce order effects that confound eye state with practice or fatigue. Future studies should counterbalance the administration order or employ separate testing sessions with adequate washout periods.

Third, our cross-sectional design precludes strong causal inferences. It remains possible that pre-existing individual differences contribute to both the choice to reside at high altitude and the observed perceptual phenotype. The language used throughout this manuscript (e.g., “associated with,” “may contribute to“) reflects this limitation. Longitudinal studies tracking individuals before and after relocation to high altitude would provide stronger evidence for experience-dependent adaptation.

Fourth, immigration duration served as a proxy for physiological adaptation, but this is a crude indicator. Individuals with the same duration of residence may exhibit substantial variability in physiological adaptation status. Future research should incorporate objective adaptation indices such as SpO2, the Lake Louise Score, or hemoglobin mass to more precisely quantify adaptation level.

Fifth, the IL-6 missing data rate (26.8%) and the marginal significance of the IL-6 association (*p* = 0.053) warrant cautious interpretation. Although Little’s MCAR test supported the missingness mechanism and supplementary analyses (Little, 1988) yielded consistent patterns, the IL-6 finding should be considered preliminary.

Sixth, the fixed-order VVIQ administration and the relatively small sample size (*N* = 112) for latent profile analysis may limit the generalizability and stability of our classification results. Larger, independent replication samples are needed.

Seventh, Our evidence is suggestive rather than confirmatory, and that the non-significant interaction may reflect limited statistical power given the unequal group sizes (*n* = 87 vs. *n* = 25).

Finally, while our analyses identified several physiological predictors, the complex interaction effects and potential hierarchical structure among inflammatory, hematological, hepatic, and autonomic factors remain to be fully characterized. Future research employing structural equation modeling or machine learning with larger samples could elucidate causal pathways and relative importance of these systems.

## 5 Conclusion

In summary, chronic high-altitude exposure associates with a reweighting of perceptual priorities, shifting the balance toward an interoception-dominant mode of processing. This adaptive strategy is characterized by a differential association pattern in attentional networks—wherein orienting attention supports internal simulation and alerting attention maintains external vigilance—and is modulated by a coordinated physiological axis involving vagal tone, erythrocytic heterogeneity, and inflammatory signaling. By moving beyond traditional metrics of processing speed or efficiency, this work reveals a core, priority-level mechanism of human adaptation to extreme hypoxia, offering a refined framework for understanding brain–body resilience in challenging environments.

## Data Availability

The raw data supporting the conclusions of this article will be made available by the authors, without undue reservation.
